# SARS-CoV-2 RBD-Specific Antibodies Induced Early in the Pandemic by Natural Infection and Vaccination Display Cross-Variant Binding and Inhibition

**DOI:** 10.3390/v14091861

**Published:** 2022-08-24

**Authors:** Melanie R. Walker, Daria Podlekareva, Stine Johnsen, Bonna Leerhøy, Cyrielle Fougeroux, Max Søgaard, Ali Salanti, Sisse Bolm Ditlev, Lea Barfod

**Affiliations:** 1 Centre for Medical Parasitology, Department of Immunology and Microbiology, Faculty of Health and Medical Sciences, University of Copenhagen, 2200 Copenhagen, Denmark; 2 Centre for Translational Research, Bispebjerg Hospital, 2400 Copenhagen, Denmark; 3 Expres2ion Biotechnologies, 2970 Hørsholm, Denmark

**Keywords:** COVID-19, SARS-CoV-2, antibody, inhibition, variants of concern

## Abstract

The development of vaccine candidates for COVID-19 has been rapid, and those that are currently approved display high efficacy against the original circulating strains. However, recently, new variants of severe acute respiratory syndrome coronavirus 2 (SARS-CoV-2) have emerged with increased transmission rates and less susceptibility to vaccine induced immunity. A greater understanding of protection mechanisms, including antibody longevity and cross-reactivity towards the variants of concern (VoCs), is needed. In this study, samples collected in Denmark early in the pandemic from paucisymptomatic subjects (*n* = 165) and symptomatic subjects (*n* = 57) infected with SARS-CoV-2 were used to assess IgG binding and inhibition in the form of angiotensin-converting enzyme 2 receptor (ACE2) competition against the wild-type and four SARS-CoV-2 VoCs (Alpha, Beta, Gamma, and Omicron). Antibodies induced early in the pandemic via natural infection were cross-reactive and inhibited ACE2 binding of the VoC, with reduced inhibition observed for the Omicron variant. When examined longitudinally, sustained cross-reactive inhibitory responses were found to exist in naturally infected paucisymptomatic subjects. After vaccination, receptor binding domain (RBD)-specific IgG binding increased by at least 3.5-fold and inhibition of ACE2 increased by at least 2-fold. When vaccination regimens were compared (two doses of Pfizer-BioNTech BNT162b2 (*n* = 50), or one dose of Oxford-AstraZeneca ChAdOx1 nCoV-19 followed by Pfizer-BioNTech BNT162b2 (ChAd/BNT) (*n* = 15)), higher levels of IgG binding and inhibition were associated with mix and match (ChAd/BNT) prime-boosting and time since vaccination. These results are particularly relevant for countries where vaccination levels are low.

## 1. Introduction

Severe acute respiratory syndrome coronavirus 2 (SARS-CoV-2) emerged in Wuhan, China at the end of 2019 and case numbers have increased at an alarming rate, resulting in one of the most challenging world-wide health crises in recent history [1]. As of June 2022, over 530 million cases of coronavirus disease (COVID-19) have been confirmed and an estimated 15 million deaths have occurred [2,3].

Over the course of the pandemic, SARS-CoV-2 variants of concern (VoC), including Alpha, Beta, Gamma, Delta, and Omicron, have emerged [4]. Many of the mutations present in these variants are located in the ~22 kDa receptor binding domain (RBD) of the spike protein, which acts as the main receptor facilitating virus entry by interacting with angiotensin-converting enzyme 2 receptor (ACE2) on host epithelial cells [5]. In SARS-CoV-2 infection, most of the protective antibodies are thought to be IgG directed towards the RBD, which has four main antibody binding regions [6,7]. Mutations in the RBD, including N501Y and A570D in the Alpha variant and K417N/T, E484K, and N501Y in the Beta and Gamma variants, have been shown to escape therapeutic mAbs, increase transmission rates and reduce susceptibility to vaccine induced immunity [8,9,10,11]. Furthermore, the now dominant Omicron variant has 15 mutations (5339D, S371L, S373P, S375F, K417N, N440K, G446S, S447N, T478K, E484A, Q493R, G496S, Q498R, N501Y, Y505H) in the RBD alone, many of which have been associated with immune evasion [12,13,14,15,16].

The development of vaccine candidates for COVID-19 has been rapid and those that are currently approved have shown substantial efficacy against the original circulating variants [17]. Due to waning immunity and the emergence of new variants, many countries have started to administer 3rd and even 4th booster doses [17,18,19,20]. However, even in countries with a high proportion of people receiving 3 doses of vaccine, infection is still a reality and can be attributed to either poor or fast-decaying vaccine-induced immunity [19]. In many less wealthy countries, vaccine doses remain scarce, resulting in low vaccination rates; in particular, first and second doses are yet to be administered [21]. Globally, priming and booster doses have been mixed and matched. This is due to both the limited availability of vaccine doses and the rare, but severe side effect of thrombosis in combination with thrombocytopenia, which has been reported after the administration of some vaccine formulations [22,23]. These vaccine combinations have been shown to be more effective when compared with the homologous prime-boost regime [24,25,26]. Additionally, heterologous booster doses are essential in countries using inactivated viral vaccines as these have low seroconversion efficacy [27]. Nevertheless, there are a limited number of studies looking at how effective these combinations are against highly mutated variants, especially Omicron [28,29,30,31]. Furthermore, these studies do not examine the heterologous prime-boost regimen of Oxford-AstraZeneca ChAdOx1 nCoV-19 (Vaxzevria^®^, Oxford, UK) followed by Pfizer-BioNTech BNT162b2 (Comirnaty^®^, Puurs, Belgium) (ChAd/BNT) [28,29,30,31]. Understanding how effective the ChAd/BNT regimen is against highly mutated variants will give us insight into whether modified or alternate vaccine platforms are needed. 

All vaccines on the market induce immune responses towards the spike protein only, which makes the vaccine efficacy especially vulnerable to changes in the epitopes within the RBD. Companies are already modifying their vaccines to compensate for these immune escape variants [32,33] and alternative vaccine platforms are being explored with the intension of inducing stronger responses with more longevity [34]. A vaccine is desperately needed that will be broadly effective against diverse SARS-CoV-2 variants. Before this can be achieved, a greater understanding of protection mechanisms, including antibody longevity and cross-reactivity towards the VoCs is needed. This knowledge would be particularly relevant for those with naturally acquired and vaccine-induced SARS-CoV-2 immunity from early in the pandemic who are presumably naïve to these variants. 

Here, samples collected in Denmark early in the pandemic from COVID-19 positive subjects with severe symptoms or paucisymptomatic subjects who were subsequently vaccinated were used to assess IgG binding and inhibition in the form of ACE2 competition against the wild-type (WT) and four SARS-CoV-2 VoCs (Alpha, Beta, Gamma, and Omicron). Antibodies induced early in the pandemic via natural infection followed by vaccination were cross-reactive and functional against all VoCs, with a reduced functionality observed for Omicron. These results are particularly relevant for countries where vaccination levels are low.

## 2. Materials and Methods

### 2.1. Subject and Sample Characteristics

Blood samples from each subject were collected in serum vacutainers and separated by centrifugation. The serum was then collected and stored at −80 °C until use. Questionnaires regarding symptoms of infection, type of work, and demographics were completed and entered into a database as previously described [35,36].

Included in this study were 57 unvaccinated previously described subjects [36] with severe symptoms (referred to here as symptomatic subjects) who were PCR positive for SARS-CoV-2 between March 2020 and June 2020 (Table 1, Figure S1). These 57 subjects included hospitalized patients and patients referred by their general practitioner [36]. These patients demonstrated objective respiratory, functional, radiological, and cognitive abnormalities [36]. All 57 subjects had samples collected between August 2020 and October 2020, with a median time since infection of 161 days (range 64–260 days). All patient characteristics have been described previously [36]. 

Paucisymptomatic subjects (people with minor or no symptoms that did not seek medical care) were recruited from Bispebjerg Hospital, Copenhagen, Denmark during screening of SARS-CoV-2 antibodies in hospital staff employed in the Capital Region of Denmark as previously described [35]. Serum was collected from 507 subjects and screened for either IgG, IgM, or IgA antibodies towards the WT spike protein (Figure S1). Among those, a total of 165 hospital staff who tested positive for IgA, IgM, or IgG in the initial screening were included in this longitudinal study and are referred to here as paucisymptomatic subjects (Table 1). All patients that participated in the initial screening had not received any SARS-CoV-2 vaccination. Initial samples were collected from April 2020 to September 2020. Follow-up samples were then collected from 66 of the 165 subjects between June 2020 and September 2020. During this time, patients may have experienced re-infection. However, screening for re-infection was not performed. Additionally, from December 2020 to June 2021, 44 of these 165 subjects were vaccinated, and post-vaccination samples were collected (Table S1). An additional 21 vaccinated healthcare workers were also recruited (Table S1). In total, 65 subjects were fully vaccinated and of these, 50 were vaccinated with two doses of Pfizer-BioNTech BNT162b2 (Comirnaty^®^) (referred to here as BNT/BNT), and 15 were vaccinated with one dose of Oxford-AstraZeneca ChAdOx1 nCoV-19 (Vaxzevria^®^) followed by Pfizer-BioNTech BNT162b2 (Comirnaty^®^) (referred to here as ChAd/BNT). 

### 2.2. Ethics Statement

Human research ethics approval was obtained for all samples from the Regional Research Ethics Committees for the Capital Region of Denmark (Protocols H-4-2013-083, H-20035553, and H-20034367) and all patients gave informed consent. All methods were performed in accordance with the relevant guidelines and regulations. 

### 2.3. Expression and Purification of Recombinant Proteins

All the antigens were synthesized by Geneart and optimized for expression in the ExpreS2 platform as previously described [34]. Briefly, Schneider-2 (ExpreS2) cells were transiently transfected using transfection reagent (ExpreS2 Insect TRx5, ExpreS2ion Biotechnologies) according to the manufacturer’s protocol. Recombinant SARS-CoV-2 antigens (RBD WT SARS-CoV-2, or its Alpha (B.1.1.7), Beta (B.1.351), Gamma (P.1) or Omicron (B1.1.529) variants, aa319-591) had an N-terminal BiP secretion signal and a C-terminal C-tag (N-RBD-EPEA-C) used for purification [34]. The ACE2 protein (aa.1–615)-C-terminal Twin-Strep-tag (Iba, GmbH) and the spike protein (aa.16-1208)-Ctag (ΔTM-ΔFurin-CoV-PP-Ctag)) were N-terminally tagged with a BiP secretion signal. 

### 2.4. Enzyme Linked Immunoassays

Microtiter 96-well plates, (Nunc Maxisorb, ThermoFisher Scientific) were coated with 2 μg/mL of antigen (spike from WT SARS-CoV-2, RBD WT SARS-CoV-2 or its Alpha (B.1.1.7), Beta (B.1.351), Gamma (P.1) or Omicron (B1.1.529) variants) and incubated overnight. Plates were washed three times with PBS-T and then blocked for one hour with blocking buffer (5% non-fat dry milk in PBS-T). The bound antigen was then incubated with human serum at a final dilution of 1:125. This was followed by a 1 h incubation with the desired detection antibody: anti-human IgG-AP (Sigma Aldrich, St. Louis, MO, USA, 1:1500), anti-human IgM-AP (Sigma Aldrich, 1:1500), or anti-human IgA-AP (Sigma Aldrich, 1:1500). Next, an incubation with 4-Nitrophenyl phosphate disodium salt hexahydrate tablets (Sigma Aldrich) dissolved in 1 × Diethanolamine Substrate Buffer (Sigma Aldrich) was performed. Color development and absorbance were measured at 405 nm. 

Plasma pools of exposed adults against each antigen were used as positive controls and to normalize plate-to-plate variability. Antibody levels were presented as arbitrary units (AU) and calculated as (OD_sample_-OD_blank_)/(OD_positive control_-OD_blank_). In order to define a true positive result, a cut-off value was calculated for each assay as the mean + 3SD of OD values in sera collected in 2013 from 10 healthy Danish donors [37,38]. 

For competition, blocking was performed with 5% w/v bovine serum albumin (BSA) (Sigma Aldrich). The bound antigen was then incubated for one hour with serum (1:25 dilution) in 1% BSA. Recombinant ACE2 was added corresponding to 90% maximal binding for the WT and each VoC (RBD WT SARS-CoV-2, 54 nM; Alpha (B.1.1.7), 4 nM; Beta (B.1.351), Gamma (P.1), 14 nM, and Omicron (B1.1.529) 54 nM). Bound ACE2 was detected using HRP conjugated strep-tactin (IBA, 1:10,000 dilution) and TMB substrate. On each plate, a plasma pool of immune serum was used as a positive control and commercially bought normal human serum (Sigma) as a negative control. ACE2 binding without antibody served as a “normal binding” control and was used to determine percent inhibition, which was calculated using the formula % inhibition = 1 − (inhibited activity)/(‘normal’ binding)] × 100, after subtraction of background. In order to define a true positive result, a cut-off value was calculated for the WT and each VoC as the mean + 3SD of inhibition values in sera collected from 10 healthy Danish donors in 2013 [37,38] (5.7%, 4.7%, 14.9%, 7.2%, and 13.7% for WT, Alpha, Beta, Gamma, and Omicron variants respectively). 

### 2.5. Statistical Analysis

Data analysis was performed and graphs created using GraphPad Prism Software (version 7.0, GraphPad, San Diego, CA, USA). To allow for direct comparisons between all RBDs tested, IgG levels were standardized and compared as level of antibody over background cut-off. For inhibition, data was normalized for the WT and each VoC using the formula normalized data = (percent inhibition − min/max − min) × 100 where min is the cut-off for each variant and max is the maximum value in the dataset. A Friedman and Dunn’s post-hoc test was then used to evaluate antibody levels and competition between the WT and the VoCs. Chi-squared (**χ^2^**) tests were performed to compare proportions. Kruskal–Wallace and Dunn’s post-hoc tests were used for comparisons between longitudinal samples. Mann–Whitney tests were used to evaluate statistical significance between two groups. A two-way ANOVA with Tukey’s post-hoc testing was applied to group wise time points. Linear regression was performed using default parameters. Throughout all analysis, statistical significance was defined as a *p* value less than 0.05 and results expressed as mean ± standard deviation (SD).

## 3. Results

### 3.1. Cross-Reactive Inhibitory Antibodies Are Detected in Subjects with Severe COVID-19 Symptoms and Paucisymptomatic Subjects

**Figure 1:**
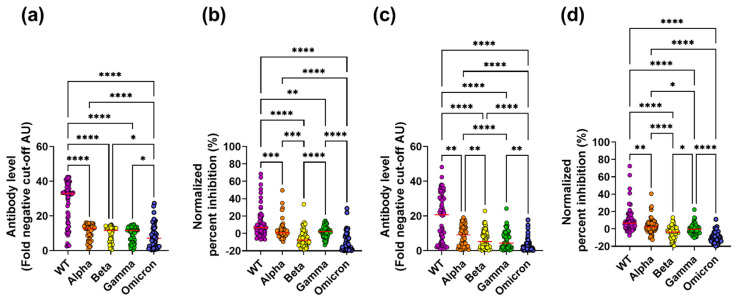
RBD-specific IgG levels and ACE2 inhibition for SARS-CoV-2 WT and four VoCs in symptomatic and paucisymptomatic subjects. RBD-specific IgG binding ((**a**,**c**) (antibody level, fold negative cut-off AU)) and ACE2 inhibition ((**b**,**d**) (normalized percent inhibition)) against the WT, Alpha, Beta, Gamma, and Omicron variants was measured in subjects with severe symptoms (**a**,**b**) and paucisymptomatic subjects (**c**,**d**). A Freidman test with Dunn post-hoc test was performed between all possible combinations (* *p* = 0.05, ** *p* = 0.01, *** *p* = 0.001 and **** *p* = 0.0001).

To investigate the potential of naturally acquired antibodies to target epitopes on the RBD, which is responsible for ACE2 binding, levels of RBD-specific IgG were determined for 57 subjects who previously had a COVID-19 infection with severe symptoms [36]. This revealed 55 of the 57 subjects (96.5%) with detectable WT RBD-specific IgG (Figure 1a). To further investigate the cross-reactive nature of these WT RBD-specific antibodies induced early in the pandemic, these 55 samples were tested on the Alpha, Beta, Gamma, and Omicron variants, all of which emerged in Denmark after October 2020, when these initial samples were collected [39,40]. This revealed 55 (100%) subjects with reactivity to the Alpha variant, 54 (98%) to the Beta and Gamma variants, and 50 (90.9%) to the Omicron variant (**χ^2^**, *p* = 0.1139, Figure S2). To allow for direct comparisons of RBD-specific IgG between the WT and the VoC, the data was standardized and expressed as a level of IgG over the background cut-off. We found detectable levels of IgG binding to the WT RBD as well as to the VoC. However, the WT variant had significantly higher RBD-specific IgG responses than the Alpha, Beta, and Gamma variants (Figure 1a). Furthermore, all variants tested had significantly higher RBD-specific IgG responses than the Omicron variant. Combined, these results not only confirmed that our IgG binding assay was robust in detecting RBD-specific IgG, but also suggest that in the RBD, cross-reactive epitopes do exist in the variant strains tested. 

To further investigate the functionality of the serum antibodies, an in vitro binding assay between ACE2 and the RBD was set up [41], which tested the ability of serum samples to block the binding of the WT and VoC RBDs to ACE2. To allow for direct comparisons of inhibitory responses between the WT and the VoC, percent inhibition was normalized. For the WT, 41 of the 55 samples (74.5%) inhibited ACE2 and WT RBD binding. Inhibitory responses differed significantly among the VoCs in terms of both the proportion of samples that inhibited (Alpha; 36/55 (65.5%), Beta; 11/55 (20%), Gamma; 37/55 (67%) and Omicron; 8/55 (14.5%), **χ^2^**; *p* < 0.0001, Figure S2) and inhibition levels (Figure 1b), with the WT having the highest response. Furthermore, inhibition was significantly positively correlated with IgG binding for the WT and the VoC (with the exception of Omicron (Figure S3, symptomatic)), indicating that RBD-specific IgG levels are a predictor of inhibition. Overall, symptomatic subjects generated cross-reactive antibodies that had the ability to block the interaction between ACE2 and the RBD.

Next, RBD-specific IgG levels were determined for the 165 paucisymptomatic subjects who were found to have spike-specific IgA, IgM, or IgG and were enrolled in this study between April 2020 and September 2020. This revealed 65 subjects (40%) with WT RBD-specific IgG (Figure 1c). Next, these 65 samples with WT RBD-specific IgG were tested on four VoCs (Alpha, Beta, Gamma, and Omicron). High proportions of RBD-specific IgG binding were observed (Alpha; 60/65 (92.3%), Beta; 57/65 (87.7%), Gamma; 56/65 (86.2%), and Omicron; 41/65 (63%), Figure S2), suggesting that paucisymptomatic subjects have RBD-specific IgG antibodies with cross-variant reactivity, similarly to subjects with severe disease. Nevertheless, the proportion of subjects with RBD-specific IgG differed significantly among the VoCs (**χ^2^**, *p* < 0.0001, Figure S2), and when levels of binding were compared to the WT, binding was significantly reduced to the four VoCs (Figure 1c). Additionally, although cross-reactivity to the Omicron variant existed, the level of Omicron binding was significantly reduced compared to the WT and the other VoCs tested. 

To investigate the blocking ability of the antibodies, serum samples that bound to the WT were tested in the ACE2/RBD inhibition assay. Of the 65 samples tested, 55 (84.6%) had antibodies capable of inhibiting the binding of the WT RBD to ACE2. Inhibitory responses differed significantly among the VoCs both in regard to proportion of samples that inhibited (Alpha; 47/55 (72.3%), Beta; 19/55 (29.2%), Gamma; 33/55 (50.8%), Omicron; 6/55 (10.9%), **χ^2^**; *p* < 0.0001, Figure S2) and inhibition levels (Figure 1d) with the VoC having lower inhibition than WT. Similar to what we had observed for the symptomatic subjects, inhibition was significantly positively correlated with RBD-specific IgG levels (Figure S3), again indicating that RBD-specific IgG levels are a predictor of ACE2 competition.

Overall, cross-reactivity was observed in paucisymptomatic subjects from early in the pandemic. 

### 3.2. Sustained Cross-Reactive Inhibitory Responses Exist in Naturally Infected Paucisymptomatic Subjects

**Figure 2:**
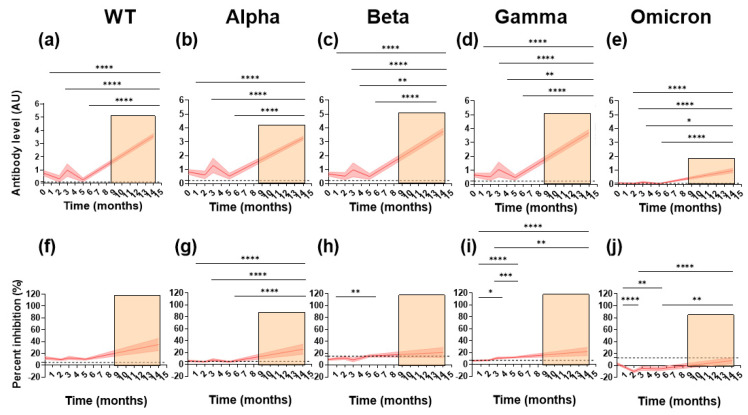
**Longitudinal levels of binding and ACE2 competition against SARS-CoV-2 WT and the VoC in paucisymptomatic subjects.** IgG binding (antibody level, AU, panels (**a**–**e**)) and ACE2 inhibition (percent inhibition, %, panels (**f**–**j**)) was measured longitudinally in paucisymptomatic subjects. The panels show cohort running means (thick red line) and their 95% confidence (red shading). Orange shading represents the time period in which subjects were vaccinated. A Kruskal–Wallace test and Dunn’s post-hoc test were performed between all possible combinations longitudinally (* *p* = 0.05, ** *p* = 0.01, *** *p* = 0.001 and **** *p* = 0.0001). Positivity cut-offs are represented by a dashed black line.

To follow the longevity of the antibody responses induced by natural infection and exposure, follow-up samples were taken from the paucisymptomatic subjects at either two, three, or five months after the first sample collection and before vaccination. All samples were tested for reactivity towards the WT RBD as well as the VoC. RBD-specific IgG levels did not significantly decrease during the five-month follow up (Figure 2a–e). Nevertheless, different patterns were observed for the individual subjects (Figure S4a–e). Reactivity decreased in the majority of individuals with initially high antibody levels (Figure S4a–e). For a few individuals, the antibody levels increased, which suggests a boost of the anti-SARS-CoV-2 immune response, potentially caused by re-exposure. The same patterns of longitudinal IgG binding were seen for the WT and the VoC. 

To understand the impact of vaccination after infection, RBD-specific IgG binding in 44 subjects who were vaccinated after the initial sampling was investigated. Vaccination occurred between December 2020 and June 2021, with samples collected in June 2021 at a median of 30 days (range; 20–92 days) after the second dose. Overall, RBD-specific IgG levels increased significantly after vaccination for the WT and the VoC when compared to the initial sampling time point and follow-up samples (Figure 2a–e). Specifically, for the WT, Alpha, Beta, and Gamma variants, IgG levels after vaccination were over 3.5 times higher than the initial sampling time points.

We next looked at the ability of the antibodies to block ACE2/RBD binding longitudinally (Figure 2f–j). Like RBD-specific IgG binding, inhibition was stable over time for the WT and Alpha variant (Figure 2f,g). Surprisingly, for the Beta and Gamma variants, inhibition increased significantly over time, suggesting a boost in immune response possibly due to SARS-CoV-2 re-exposure (Figure 2h,i). Inhibition decreased significantly for the Omicron variant, indicating limited cross-reactivity over time. An increase in inhibition was observed after vaccination. When compared to the initial sampling time point, this increase in inhibition was at least 2-fold for all variants. Nevertheless, this was only significant for the Alpha, Gamma, and Omicron variants (Figure 2g,i,j).

Together, these results indicate that longitudinal cross-variant RBD-specific IgG binding and inhibition is sustained for up to five months. Furthermore, vaccination generates high cross-reactive inhibitory antibody responses in the majority of subjects. 

### 3.3. Subjects with ChAd/BNT Prime-Boost Vaccination Have Higher Inhibition and IgG Binding against the WT and the VoC Than Those with BNT/BNT Prime-Boost

In Denmark, where this study was conducted, vaccination commenced in December 2020 with the mRNA vaccines Pfizer-BioNTech BNT162b2 (Comirnaty^®^) and Moderna 1273 (Spikevax^®^), and with adeno-vectored vaccine, Oxford-AstraZeneca ChAdOx1 nCoV-19 (Vaxzevria^®^). Due to the rare but severe side effect of thrombosis in combination with thrombocytopenia, all vaccinations with Oxford-AstraZeneca ChAdOx1 nCoV-19 (Vaxzevria^®^) were paused on 11 March 2021, and as of April 2021, withdrawn from the Danish national vaccination program [22,42]. At that time, many healthcare workers had been vaccinated with only one dose of Oxford-AstraZeneca ChAdOx1 nCoV-19 (Vaxzevria^®^) (referred to here as ChAd) and the subsequent advice from the Danish Health Authority (Sundhedsstyrelsen, SST) was to wait at least 60 days before receiving a Pfizer-BioNTech BNT162b2 (Comirnaty^®^) booster dose (referred to here as BNT) [42,43]. 

Further studies are needed to determine how effective the ChAd/BNT vaccination combination is against highly mutated variants, such as the Omicron variant. Thus, we wanted to compare IgG binding and ACE2 inhibition against the VoC between Danish healthcare workers vaccinated with the ChAd/BNT combination and those that received homologous BNT prime-boost (referred to here as BNT/BNT). Due to saturation of vaccinated samples (Figure S4a–e), in our RBD-specific IgG binding ELISA, samples were diluted and tested at 1:1250. All fully vaccinated subjects (*n* = 65) were divided into those with a ChAd dose, followed by a BNT dose (*n* = 15), and were compared to those with two BNT doses (*n* = 50) for binding and competition. It must be taken into consideration that subjects in the ChAd/BNT group had a median time between vaccinations of 76 days (range; 65–89), whereas subjects in the BNT/BNT group had a median time between vaccinations of 30 days (range; 20–52) (Mann–Whitney, *p* < 0.0001). This difference in time between vaccinations is due to advice from the Danish Health Authority at the time [42,43]. 

**Figure 3:**
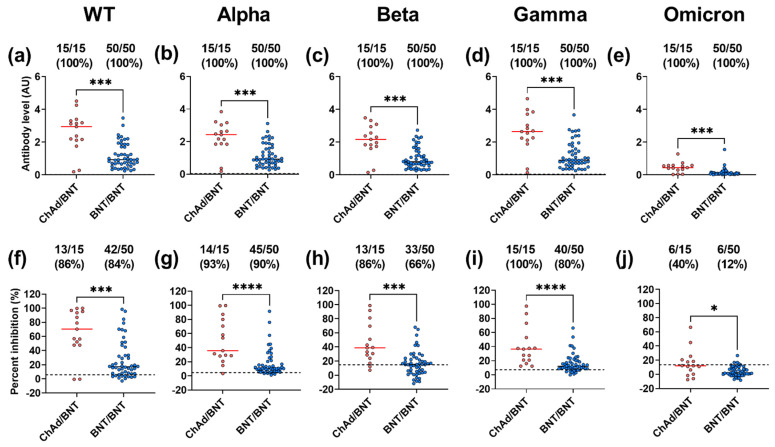
**IgG binding and ACE2 competition in vaccine groups to SARS-CoV-2 WT and the VoCs.** Levels (AU, mean with SD) of IgG (**a**–**e**), and ACE2 competition (percent inhibition) (**f**–**j**) were measured between subjects in the ChAd/BNT group (red) and those in the BNT/BNT group (blue) against SARS-CoV-2 VoC WT, Alpha, Beta, Gamma, and Omicron. A Mann–Whitney test was performed between combinations of the two vaccine groups (* *p* = 0.05, *** *p* = 0.001, **** *p* = 0.0001). Positivity cut-offs are represented by a dashed black line. For each vaccine group, the number of positive individuals, total number of individuals tested, and percent reactivity is shown at the top of each panel.

Interestingly, all vaccinated individuals in all groups had antibodies exhibiting RBD-specific binding to all variants (Figure 3a–e). Furthermore, a large proportion (>65%) of vaccinated samples from both groups inhibited WT, Alpha, Beta, and Gamma variant binding to ACE2 (Figure 3f–i). Additionally, although the proportion of vaccinated samples from the ChAd/BNT and BNT/BNT groups that inhibited Omicron was 40% and 12%, respectively, inhibition was still present (Figure 3j). 

For the WT and all the VoCs, those in the ChAd/BNT had significantly higher binding and competition than those in the BNT/BNT group (Figure 3a–j). Although timing between doses cannot be ruled out as a cause of significance, these results together indicate that individuals vaccinated at the start of the pandemic with ChAd/BNT have a robust antibody response that is cross-reactive against the VoC.

### 3.4. Vaccine Induced RBD-Specific IgG Binding and Competition Decreases over Time for the WT and the VoC

**Figure 4:**
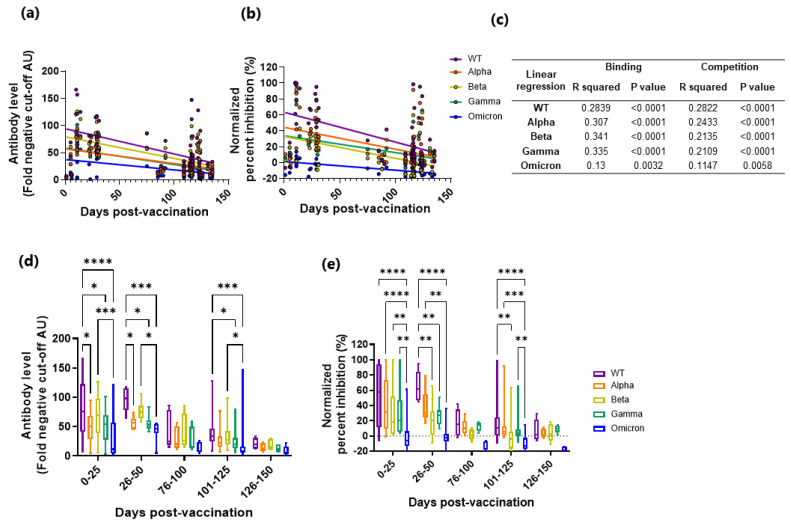
IgG binding and ACE2 competition measured against SARS-CoV-2 WT and the VoCs in vaccinated subjects stratified as days post-vaccination. Levels (fold negative cut-off) of IgG (**a**,**d**), and ACE2 competition (normalized percent inhibition, (**b**,**e**)) were measured in vaccinated subjects as days post-vaccination against the WT and the VoC (WT, purple; Alpha, orange; Beta, yellow; Gamma, green; Omicron, blue). Linear regression (**a**). IgG levels and b. ACE2 competition) was fitted and linear regression analysis performed (**c**). The data was also stratified into time windows of 25 days and responses measured between the WT and the VoC by two-way ANOVA ((**d**). IgG levels and (**e**). ACE2 competition, * *p* = 0.05, ** *p* = 0.01, *** *p* = 0.001 and **** *p* = 0.0001).

Breakthrough infections are now a reality, with some studies attributing this to fast decaying vaccine induced immunity [19] and others to immune escape by the VoC [14,15]. Here we wanted to determine antibody binding and inhibition longevity towards the VoC after vaccination. All fully vaccinated subjects (*n* = 65) were divided into days post-vaccination (median; 115 days, range; 1–135 days) and analyzed for IgG binding and competition. IgG binding and competition decreased significantly over time for the WT and the VoC (Figure 4a–c). 

In order to examine if the earlier responses were more potent against the WT, the data were stratified into time windows (Figure 4d,e, Table S2). In general, the WT had significantly higher binding and competition over time when compared to the VoC. Like the symptomatic and paucisymptomatic subjects’ samples, inhibition was significantly positively correlated with IgG binding for the WT and the VoC (Figure S3), again indicating that RBD-specific IgG levels are a predictor of ACE2 competition. Combined, these results indicate that after vaccination, antibody binding and inhibition wane, but to a lesser degree against the WT. 

## 4. Discussion

In this study, we describe cross-reactive inhibitory antibodies sampled from naturally infected symptomatic and paucisymptomatic subjects collected early in the pandemic. IgG binding to the WT RBD and the VoC RBDs was sustained for up to five months after infection, whereas longitudinal analysis revealed that inhibitory responses were variant dependent. Upon vaccination, VoC RBD-specific binding, and inhibition of ACE2 increased; however, higher levels of both binding and inhibition were associated with ChAd/BNT prime-boosting and time since vaccination. 

Throughout the study, higher IgG reactivity and inhibition towards WT RBD were found when compared to the VoC in both symptomatic and paucisymptomatic subjects. These results are consistent with previous pre-clinical studies comparing antibodies directed towards the WT spike protein to those generated against the VoC [12,14,31,44,45]. Furthermore, the samples in this study were collected prior to the appearance of the VoC, and therefore naturally acquired antibodies would be directed towards the WT strain. Nevertheless, inhibitory cross-reactive antibodies were observed in agreement with previously published results [44] and indicates that the maturation of memory B cells in subjects previously infected with SARS-CoV-2 results in a broad range of SARS-CoV-2 specific antibodies [45,46,47]. 

Longitudinal analysis indicated no significant decrease in RBD-specific IgG binding for up to five months after the first sampling time point for the WT or any of the VoCs tested. It must be noted that for the WT, there was a trend of decreasing IgG binding. However, this was not significant. The results here are supported by previous studies testing IgG against the RBD which show that in the absence of vaccination, antibody levels remain relatively stable for 6–12 months, peaking around four months after onset and then slowly decreasing [44,46]. This could explain the trend of decreasing IgG levels towards the WT protein, where responses could be starting to wane. 

Cross-reactivity of ACE2-RBD binding inhibition was observed longitudinally for all the VoCs tested. Additionally, responses to the WT and Alpha variant were sustained over the 5-month period, coinciding with the RBD-specific IgG binding observed here and similar to previously published results [44,46]. However, an increase was observed for the Beta and Gamma variants despite these variants not circulating in Denmark at the time of sample collection [40]. We propose that this increase is due to re-infection and subsequent boosting with the WT, which amplifies existing antibodies that target conserved epitopes on the RBD [48,49]. Furthermore, for the Omicron variant, limited inhibition was observed longitudinally, indicating immune escape and/or reduced affinity. These results are in accordance with other recent findings where Omicron was the most resistant to neutralization after vaccination [50,51]. 

Vaccination improved RBD-specific binding for the WT and the VoC when compared to natural infection and increased inhibition for the Alpha, Gamma, and Omicron variants but not the WT and Beta variants. As the naturally infected samples here were collected before the occurrence of the VoC, these subjects were likely infected with the WT and thus these results are likely due to high natural autologous responses.

Although vaccination increased binding and inhibition when compared to naturally infected samples, when stratified as days since vaccination, levels of binding and inhibition decreased significantly over time for the WT and the VoC. These results indicate waning immunity against the WT and the VoC after vaccination, as previously observed [21,52,53,54]. Nevertheless, there was RBD-specific binding and inhibition towards the WT and the VoC which indicates that even two doses of vaccine can elicit inhibitory antibodies against the currently circulating, highly mutated Omicron variant and is therefore important for cross-variant protection. 

Similar to previous studies, different vaccine platform prime-boosting resulted in higher inhibition than the BNT/BNT [25,26,55]. To expand on these studies, cross-reactivity was tested against four VoCs, including the currently circulating Omicron variant. It was observed that all vaccinated samples bound to the WT and the VoCs, including the Omicron variant. Furthermore, a high proportion of these samples were inhibitory. For the WT and VoC, IgG binding and ACE2 competition were higher in individuals that received the ChAd/BNT. These results are highly relevant as many countries where Omicron is currently circulating have received many different vaccine platforms and are yet to receive booster doses [21]. Notably, due to advice from the Danish Health Service at the time, subjects receiving the ChAd/BNT vaccine had a longer time between doses than those receiving BNT/BNT. Studies have shown that a longer time between doses results in stronger inhibition [55,56]. Thus, the possibility cannot be ruled out that the stronger responses seen here are due to time between vaccinations.

RBD-specific IgG binding and the degree of RBD/ACE2 inhibition correlated for all groups tested, suggesting that RBD-specific IgG antibody levels are a predictor of ACE2 inhibition. This is in accordance with previous findings where levels of RBD-specific IgG binding and ACE2 competition were also correlated [57,58]. Furthermore, RBD-specific IgG and neutralization have been shown to correlate [44]. Therefore, the presence of RBD-specific IgG may indicate the presence of neutralizing activity for most samples. Neutralizing antibodies remain the main correlate of protection, and although the ACE2 competition assay is a pseudo-neutralization assay, it does not specifically measure neutralization. However, ACE2 competition assays have been shown to correlate with neutralization activity [41,58].

Limitations of this study include the small number of participants and the small number of samples tested longitudinally. In future studies, a larger cohort with regular longitudinal sampling, especially after vaccination, would yield more concrete results. Additionally, due to the ongoing pandemic, subjects may have experienced re-infection during the period in which samples were collected. This possible re-infection could lead to a more potent antibody response longitudinally. In future work this could be avoided with routine PCR or rapid testing, which would ensure re-infection does not go undetected. Finally, other antibody-mediated effector mechanisms or T cell functions were not measured. Understanding these responses is important as it will give more insight into vaccine efficacy and clinical outcomes.

Overall, these results indicate that antibodies generated early in the pandemic through vaccination and natural infection are cross-reactive against the WT and VoC and can inhibit the interaction between ACE2 and the RBD. Additionally, this study supports previous findings that a ChAd/BNT prime-boost regimen may provide higher protection against more highly mutated variants. Nevertheless, there was reduced functionality towards the Omicron variant, indicating that vaccines will need to be modified or alternate vaccine platforms used to induce stronger responses.

## Figures and Tables

**Table 1:** Subject characteristics.

	Paucisymptomatic	Symptomatic		
***N***	**165**	**57**		
Age, median years (range)	39 (23–72)	53 (22–80)		
Female, *n* (%)	136 (83)	31 (54)		
	**BNT/BNT**	**ChAd/BNT**	**P**	**Test**
***n* ^1^**	**50**	**15**		
Age, median years (range)	44 (23–75)	40 (21–59)	0.827	Mann–Whitney
Female, *n* (%)	39 (78)	13 (87)	0.09	Fisher’s exact

**^1^** Vaccinated subjects include both paucisymptomatic subjects (*n* = 44) and additional vaccinated healthcare workers (*n* = 21) (See Figure S1 and Table S1).

## Data Availability

Not applicable.

## Supplementary Materials

The following supporting information can be downloaded at: https://www.mdpi.com/article/10.3390/v14091861/s1, Figure S1: Schematic diagram of the study design.; Figure S2: Proportion of subjects with RBD-specific IgG binding and inhibition; Figure S3: Relationship between ACE2 competition (percent inhibition) and IgG binding (antibody level (AU); Figure S4: Longitudinal levels of binding and ACE2 competition against SARS-CoV-2 WT and the VoC in paucisymptomatic subjects; Table S1: Vaccinated subject characteristics; Table S2: Statistical significance of binding and competition stratified by days post-vaccination.
