# Supplementary material for: SARS-CoV-2 RBD-Specific Antibodies Induced Early in the Pandemic by Natural Infection and Vaccination Display Cross-Variant Binding and Inhibition
Source: Viruses. 2022 Aug 24;14(9):1861. doi: 10.3390/v14091861 (PMC9503696; doi:10.3390/v14091861)
Supplement: Supplementary file 1 [file viruses-14-01861-s001.zip › viruses-1838888-supplementary.pdf]

Supplementary information

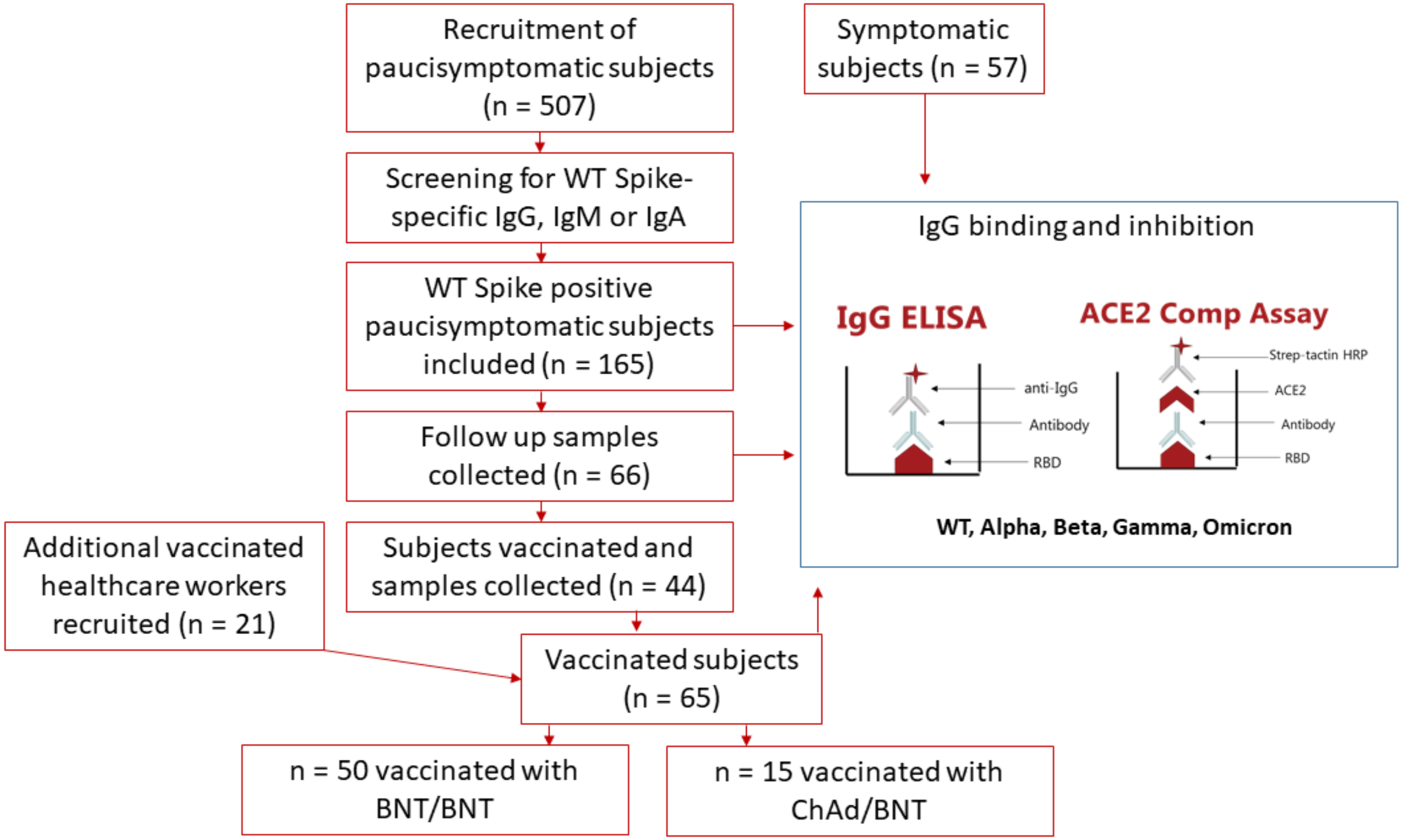

Figure S1. Schematic diagram of the study design.

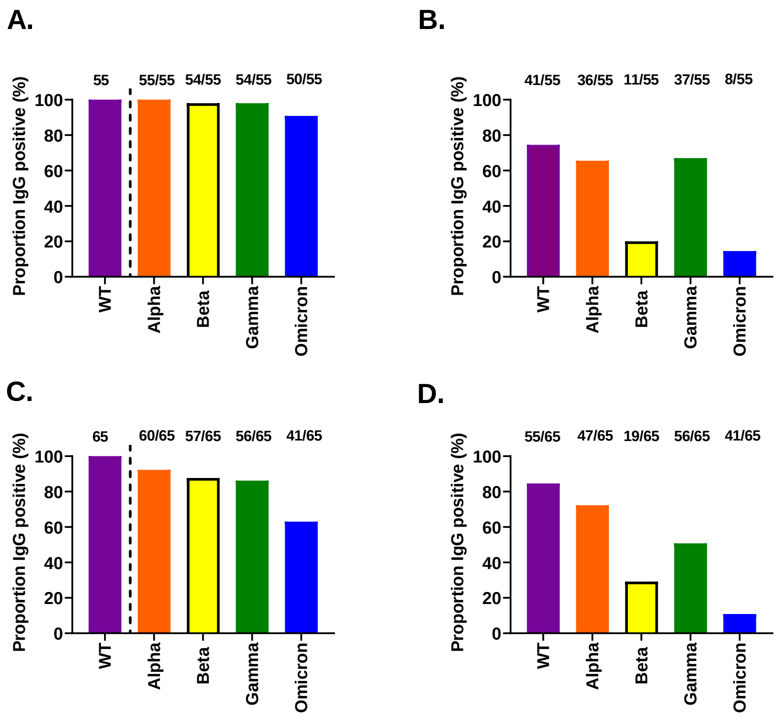

Figure S2. Proportion of subjects with RBD-specific IgG binding and inhibition. Samples with WT RBD-specific IgG (dotted line) were tested on four VoC. The proportion of binding (A, C) and inhibition (B, D) against the WT and VoC in symptomatic (A, B) and paucisymptomatic (C, D) subjects is shown. For each group, the number of positive individuals and total number of individuals tested is shown at the top of each panel.

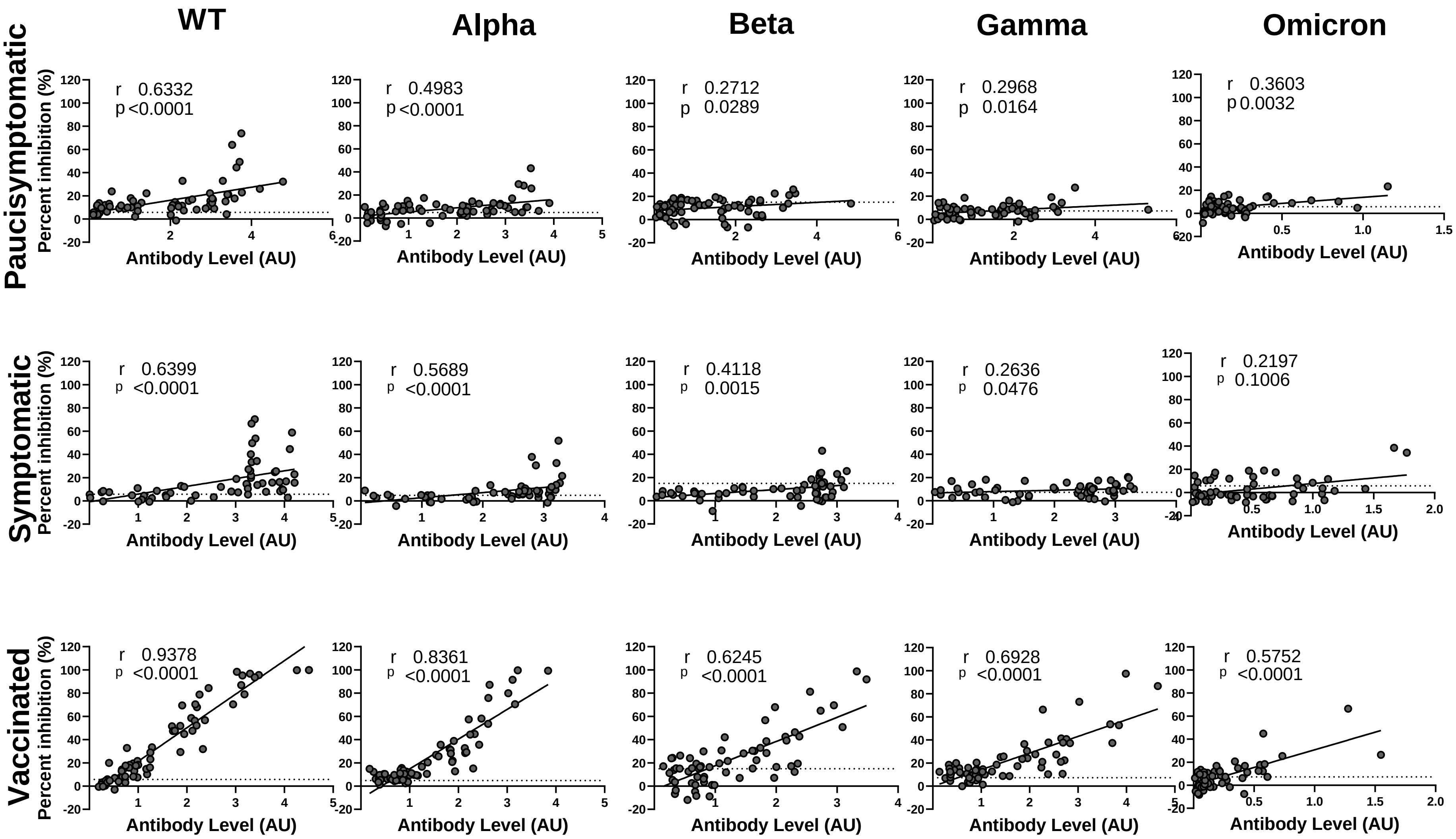
WT
Alpha
Beta
Gamma
Omicron
Paucisymptomatic
Symptomatic
Vaccinated
Percent inhibition (%)
Antibody Level (AU)
r 0.6332
p <0.0001
r 0.4983
p <0.0001
r 0.2712
p 0.0289
r 0.2968
p 0.0164
r 0.3603
p 0.0032
r 0.6399
p <0.0001
r 0.5689
p <0.0001
r 0.4118
p 0.0015
r 0.2636
p 0.0476
r 0.2197
p 0.1006
r 0.9378
p <0.0001
r 0.8361
p <0.0001
r 0.6245
p <0.0001
r 0.6928
p <0.0001
r 0.5752
p <0.0001

Figure S3. Relationship between ACE2 competition (Percent inhibition) and IgG binding (Antibody level (AU) specific for WT, Alpha, Beta, Gamma and Omicron variants, were measured by linear regression (black line) in paucisymptomatic subjects, symptomatic subjects and vaccinated subjects. Negative cut-offs for each antigen are represented by a dashed line.

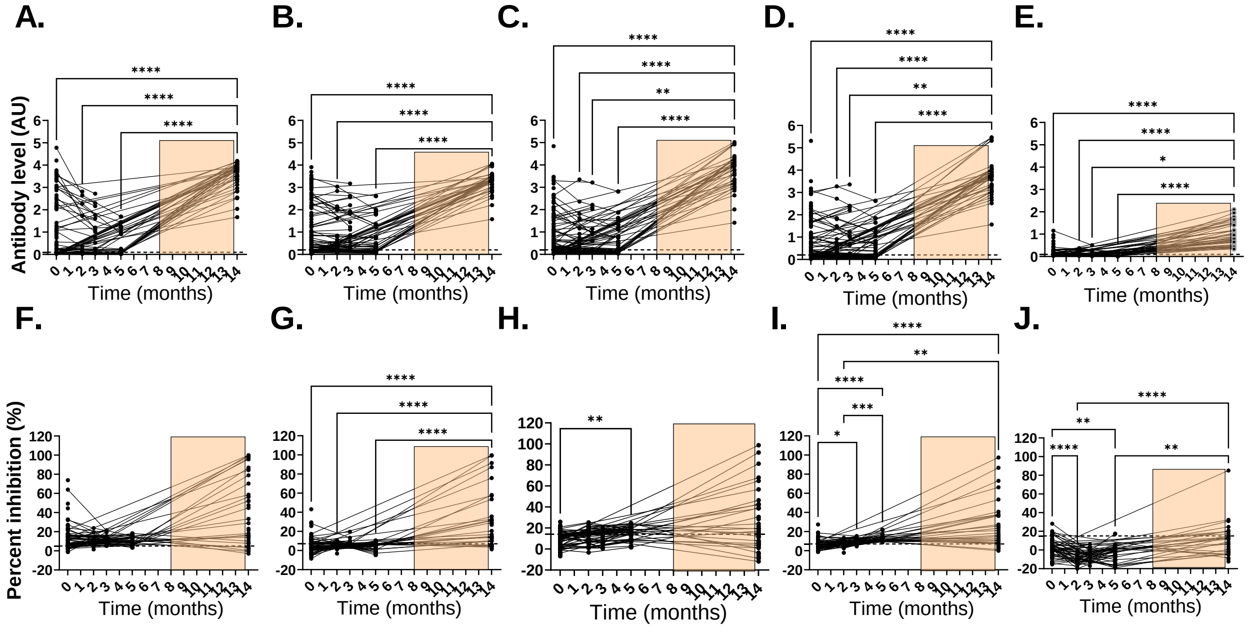

Figure S4. Longitudinal levels of binding and ACE2 competition against SARS-CoV-2 WT and the VoC in paucisymptomatic subjects. IgG binding (Antibody level, AU, Panels A-E,) and ACE2 inhibition (Percent inhibition, %, Panels F-J) against WT (A, F), Alpha (B, G), Beta (C, H), Gamma (D, I), and Omicron (E, J) variants was measured longitudinally in paucisymptomatic subjects. The panels show temporal changes in individual subjects from the first time point (time, months). Orange shading represents the time period in which subjects were vaccinated. A Kruskal-Wallace test and Dunn's post-hoc test were performed between all possible combinations longitudinally (**p* = 0.05, ** *p* = 0.01, *** *p* = 0.001, **** *p* = 0.0001). Positivity cut-offs are represented by a dashed black line.

Table S1. Vaccinated subject characteristics

	Paucisymptomatic	Healthcare workers	*p*	Test
N	**44**	**21**		
Vaccination to sampling median days (range)	116 (1 - 132)	75 (5 - 135)	NS[1]	Mann-Whitney
Time between 1st and 2nd vaccination median days (range)	31 (20 - 82)	31 (21 - 89)	NS	Mann-Whitney
ChAd/BNT heterologous boost, *n* (%)	10 (23)	4 (19)	NS	Fishers exact
Age, median years (range)	42 (24 - 64)	40 (21 - 75)	NS	Mann-Whitney
Female, *n* (%)	39 (78)	13 (87)	NS	Fishers exact

[1]NS = not significant

Table S2. Statistical significance of binding and competition stratified by days post-vaccination

	Days 0-25		Days 26-50		Days 76-100		Days 101-125		Days 126-150	
	**Binding**	**Competition**	**Binding**	**Competition**	**Binding**	**Competition**	**Binding**	**Competition**	**Binding**	**Competition**
WT vs. Alpha	*[2]	ns[1]	*	ns	ns	ns	ns	ns	ns	ns
WT vs. Beta	ns	ns	ns	**[3]	ns	ns	ns	**	ns	ns
WT vs. Gamma	*	ns	*	**	ns	ns	*	ns	ns	ns
WT vs. Omicron	****[5]	****	***	****	ns	ns	***	****	ns	ns
Alpha vs. Beta	ns	ns	ns	ns	ns	ns	ns	ns	ns	ns
Alpha vs. Gamma	ns	ns	ns	ns	ns	ns	ns	ns	ns	ns
Alpha vs. Omicron	ns	****	ns	**	ns	ns	ns	***	ns	ns
Beta vs. Gamma	ns	ns	ns	ns	ns	ns	ns	ns	ns	ns
Beta vs. Omicron	***[4]	**	*	ns	ns	ns	*	ns	ns	ns
Gamma vs. Omicron	ns	**	ns	ns	ns	ns	ns	**	ns	ns

[1]NS = not significant, [2]*$p = 0.05$, [3]**$p = 0.01$, [4]***$p = 0.001$, [5]****$p = 0.0001$
